# Mitofusin 2 Is Essential for IP_3_-Mediated SR/Mitochondria Metabolic Feedback in Ventricular Myocytes

**DOI:** 10.3389/fphys.2019.00733

**Published:** 2019-07-18

**Authors:** Lea K. Seidlmayer, Christine Mages, Annette Berbner, Petra Eder-Negrin, Paula Anahi Arias-Loza, Mathias Kaspar, Moshi Song, Gerald W. Dorn, Michael Kohlhaas, Stefan Frantz, Christoph Maack, Brenda Gerull, Elena N. Dedkova

**Affiliations:** ^1^Department of Internal Medicine, Cardiology, University Hospital Würzburg, Würzburg, Germany; ^2^Comprehensive Heart Failure Center, University of Würzburg, Würzburg, Germany; ^3^Department of Internal Medicine, Center for Pharmacogenomics, Washington University School of Medicine, St. Louis, MO, United States; ^4^Department of Pharmacology, School of Medicine, University of California, Davis, Davis, CA, United States; ^5^Department of Molecular Biosciences, School of Veterinary Medicine, University of California, Davis, Davis, CA, United States

**Keywords:** mitofusin 2, IP_3_ signaling, SR/mitochondria metabolic feedback, mitochondrial mRyR1, ATP generation, endothelin-1, Mfn2 KO mice

## Abstract

**Aim:** Endothelin-1 (ET-1) and angiotensin II (Ang II) are multifunctional peptide hormones that regulate the function of the cardiovascular and renal systems. Both hormones increase the intracellular production of inositol-1,4,5-trisphosphate (IP_3_) by activating their membrane-bound receptors. We have previously demonstrated that IP_3_-mediated sarcoplasmic reticulum (SR) Ca^2+^ release results in mitochondrial Ca^2+^ uptake and activation of ATP production. In this study, we tested the hypothesis that intact SR/mitochondria microdomains are required for metabolic IP_3_-mediated SR/mitochondrial feedback in ventricular myocytes.

**Methods:** As a model for disrupted mitochondrial/SR microdomains, cardio-specific tamoxifen-inducible mitofusin 2 (Mfn2) knock out (KO) mice were used. Mitochondrial Ca^2+^ uptake, membrane potential, redox state, and ATP generation were monitored in freshly isolated ventricular myocytes from Mfn2 KO mice and their control wild-type (WT) littermates.

**Results:** Stimulation of ET-1 receptors in healthy control myocytes increases mitochondrial Ca^2+^ uptake, maintains mitochondrial membrane potential and redox balance leading to the enhanced ATP generation. Mitochondrial Ca^2+^ uptake upon ET-1 stimulation was significantly higher in interfibrillar (IFM) and perinuclear (PNM) mitochondria compared to subsarcolemmal mitochondria (SSM) in WT myocytes. Mfn2 KO completely abolished mitochondrial Ca^2+^ uptake in IFM and PNM mitochondria but not in SSM. However, mitochondrial Ca^2+^ uptake induced by beta-adrenergic receptors activation with isoproterenol (ISO) was highest in SSM, intermediate in IFM, and smallest in PNM regions. Furthermore, Mfn2 KO did not affect ISO-induced mitochondrial Ca^2+^ uptake in SSM and IFM mitochondria; however, enhanced mitochondrial Ca^2+^ uptake in PNM. In contrast to ET-1, ISO induced a decrease in ATP levels in WT myocytes. Mfn2 KO abolished ATP generation upon ET-1 stimulation but increased ATP levels upon ISO application with highest levels observed in PNM regions.

**Conclusion:** When the physical link between SR and mitochondria by Mfn2 was disrupted, the SR/mitochondrial metabolic feedback mechanism was impaired resulting in the inability of the IP_3_-mediated SR Ca^2+^ release to induce ATP production in ventricular myocytes from Mfn2 KO mice. Furthermore, we revealed the difference in Mfn2-mediated SR-mitochondrial communication depending on mitochondrial location and type of communication (IP_3_R-mRyR1 *vs.* ryanodine receptor type 2-mitochondrial calcium uniporter).

## Introduction

Heart failure (HF) is one of the most common chronic conditions associated with aging. Despite advances in HF treatment, it has a poor prognosis with a steady increase in mortality and incidence in the aging population (Lloyd-Jones et al., 2002; Nieminen et al., 2006). Current treatments of HF rely almost entirely on altering the neurohormonal milieu, using agents such as angiotensin converting enzyme (ACE) inhibitors, angiotensin receptor blockers, and beta-adrenergic blockers to improve cardiac function and reduce mortality. Importantly, there are no proven interventions that are directly targeted toward maintaining cardiac function and myocyte survival. The pathogenesis of HF is the result of numerous changes in the whole organism with striking subcellular modifications especially at the level of mitochondria: the number of cristae of the inner mitochondrial membrane (IMM) is reduced, and due to subcellular changes, the contact sites of mitochondria and the sarcoplasmic reticulum (SR) are significantly diminished. Depending on the type of cardiomyopathy, both decreased respiratory chain activity due to impaired mitochondrial complexes I and III (Ventura-Clapier et al., 2011; Thai et al., 2018) or increased respiratory chain activity due to increased expression of uncoupling proteins (Sebastiani et al., 2007), could lead to increased proton leak and decline in ATP generation. The heart has the highest energy demand by weight of any organ in the body, and its function fails within minutes if mitochondrial ATP production is interrupted. Therefore, addressing mitochondrial dysfunction and decrease in mitochondrial ATP generation may represent an effective treatment for HF directly targeting the key features in its pathogenesis.

An increase in mitochondrial calcium (Ca^2+^) concentration ([Ca^2+^]_m_) is thought to be one of the major factors responsible for the activation of Ca^2+^-dependent dehydrogenases eventually leading to stimulation of the oxidative phosphorylation and ATP generation (Balaban, 2009). Recent studies utilizing mitochondrial Ca^2+^ uniporter (MCU) knockout (KO) mice (Pan et al., 2013; Kwong et al., 2015) questioned this accepted dogma since mitochondrial bioenergetics and ATP generation were not significantly compromised in those animals. Only when cardiac myocytes were challenged with the beta-adrenergic agonist isoproterenol (ISO), MCU KO myocytes were not able to match ATP production and respiratory rates in response to an increased work load (Kwong et al., 2015). These data indicate that although MCU is considered the major pathway for mitochondrial Ca^2+^ uptake in cardiac myocytes, other pathways located in the inner mitochondrial membrane (IMM) may compensate for the lack of MCU and play a role in the regulation of mitochondrial Ca^2+^ uptake and ATP generation. The mitochondrial ryanodine receptor (mRyR1) was proposed to be an alternative mitochondrial Ca^2+^ uptake pathway in cardiac myocytes (Beutner et al., 2005; Jakob et al., 2014) while the physiological function and relevance of this pathway are still controversial. Recently, we were able to demonstrate that Ca^2+^ released from the sarcoplasmic reticulum (SR) *via* the inositol 1,4,5-trisphosphate receptor (IP_3_R) is taken up into the mitochondrial matrix *via* the mRyR1 (Seidlmayer et al., 2016), where it activates ATP production. This IP_3_-mediated mitochondria/SR feedback is essential for the adaptation of mitochondrial metabolism to chronic stress as induced by endothelin-1 (ET-1) or angiotensin (Ang II).

Ca^2+^ release from intracellular stores is the essential signal generated by electrical activation in cardiomyocytes, which is required to initiate cell contraction in the process known as excitation-contraction coupling (ECC). To release Ca^2+^ from the SR, cardiac myocytes express two different Ca^2+^ release channels: (1) the ryanodine receptor type 2 (RyR2), which is Ca^2+^ sensitive and releases Ca^2+^ from the SR during ECC upon Ca^2+^ entrance through the voltage-gated L-type Ca^2+^ channels and (2) the inositol 1,4,5-trisphosphate (IP_3_) receptor (IP_3_R), which releases Ca^2+^ after binding of IP_3_ independently from ECC. IP_3_ is cleaved from phosphatidylinositol-4,5-bisphosphate (PIP_2_) by phospholipase C when hormones like ET-1 or Ang II bind to their membrane bound G-protein-coupled receptors. Although IP_3_Rs are less abundant in ventricular myocytes compared to RyR2s, their activation results in measurable Ca^2+^ increase during diastole and systole creating areas with highly, localized concentrations of Ca^2+^, known as microdomains. These microdomains are typically formed at the junction between the SR and mitochondria resulting in elevation of the cytosolic Ca^2+^ from 0.1 to ~30 μM, which leads to the activation of mitochondrial Ca^2+^ uptake (Szalai et al., 2000; Dedkova and Blatter, 2008; Dedkova et al., 2013; Dorn and Maack, 2013).

Cardiac myocytes display a highly organized structure: mitochondria, SR, and the contractile apparatus are tightly packed. Mitochondria occupy about 40% of the myocytes volume (Zhou et al., 1998). Based on the proximity of mitochondria to the Ca^2+^ release sites, mitochondria are divided into subsarcolemmal (SSM), interfibrillar (IFM) and perinuclear mitochondria (PNM). All three subgroups differ in enzymatic activity (Palmer et al., 1977; Matlib et al., 1978; McMillin-Wood et al., 1980) and shape (Fawcett and McNutt, 1969; Lukyanenko et al., 2009). SSM are located underneath the sarcolemma, PNM are located around nuclei, while IFM are localized between the myofilaments where they are in close contact to the Ca^2+^ release sites in the junctional SR (Chikando et al., 2011).

In addition to this spatial relationship, mitochondria and the SR are linked physically by electron dense structures called tethers (Csordás et al., 2006). In mammalian cells, these tethers are formed by protein complexes containing IP_3_R (Szabadkai et al., 2006) and mitofusins, mainly containing mitofusin 1 (Mfn1) and mitofusin 2 (Mfn2). Despite both molecules are homologues, Mfn1 mainly mediates mitochondrial fusion (Cipolat et al., 2004), whereas Mfn2 plays an important role in mitophagy (Schrepfer and Scorrano, 2016), controls the shape of the endoplasmic reticulum, and tethers mitochondria and the SR mechanically to each other to ensure the proper communication between the two organelles (de Brito and Scorrano, 2008). This latter function of Mfn2 has been challenged by Filadi et al. (2015) who suggested that Mfn2-tethers actually keep mitochondria far away from the SR to prevent mitochondrial Ca^2+^ overload. In a series of experiments, they demonstrated that KO of Mfn2 in cultured cells improved the communication between the two organelles, suggesting that Mfn2 is a negative regulator of tethering. Critical re-evaluation of Mfn2 function, however, confirmed the original discovery from Scorrano’s group that Mfn2 tethers serve to mediate the proper transfer of Ca^2+^ from the SR to mitochondria (Naon et al., 2016). In non-excitable cells, it has been shown that IP_3_Rs-mediated Ca^2+^ tunneling to mitochondria is an essential modulator of cell bioenergetics and ATP production (Cárdenas et al., 2010). However, the role of Mfn2 in IP_3_Rs-mediated Ca^2+^ tunneling to mitochondria and energetics in cardiac myocytes has not been evaluated yet.

In the present study, we aimed to examine the role of Mfn2 in the IP_3_Rs-mediated SR/mitochondria metabolic feedback mechanism described above. The hypothesis is that an intact linkage of SR and mitochondria is required for basal metabolic control of the IP_3_-mediated Ca^2+^ release in cardiac myocytes. Our hypothesis was tested using the cardiac-specific, tamoxifen-induced knockout of Mfn2 (Mfn2 KO). We found that when the physical link between SR and mitochondria by Mfn2 was disrupted, the SR/mitochondrial metabolic feedback mechanism was impaired resulting in the inability of the IP_3_-mediated SR Ca^2+^ release to induce ATP production in ventricular myocytes from Mfn2 KO mice. We further revealed the difference in Mfn-2 mediated SR-mitochondrial communication depending on mitochondrial location and type of receptor (IP_3_R *vs.* Ryanodine receptor), which can explain the existing controversy in the field.

## Materials and Methods

### Animal Model

Mfn2^loxp/loxp/cre^ (Mfn2 KO) mice were obtained from the laboratory of Prof. Gerald W. Dorn, II in St. Louis, Missouri, USA. For induction of Cre-mediated deletion of Mfn2, mice were fed 20 mg tamoxifen/kg body weight/day for 7 days. Littermates of Mfn2 KO mice (heterozygous for Mfn2) were used as control animals and designated as wild-type (WT) group. All experimental procedures were in accordance with the Guide for the Care and Use of Laboratory Animals published by the US National Institutes of Health (8th Edition, 2011) and approved by the University of Würzburg Institutional Animal Care and Use Committee.

### Cell Isolation

Adult left ventricular myocytes were isolated from 10- to 16-week-old Mfn2 KO mice and their healthy littermates. Mice were anesthetized with isoflurane and sacrificed by cervical dislocation. The excised heart was mounted to a Langendorff perfusion system and retrogradely perfused with Ca^2+^-free washing solution followed by enzyme solution containing Liberase TH (Research Grade, Roche, Basel, Switzerland) as described previously (Seidlmayer et al., 2016). Isolated myocytes were kept in Tyrode solution containing 1 mM Ca^2+^.

### Immunoblot Analysis

Left ventricular tissue was homogenized in ice-cold lysis buffer (50 mM Tris pH 7.5, 1 mM EDTA, 1 mM EGTA, 10% glycerol, 1% triton X-100, 50 mM NaF, 5 mM Na_4_P_2_O_7_, 1 mM Na_3_VO_4_, 1 mM DTT, protease inhibitor cocktail), and protein content was determined using the BCA assay. About 30 μg of protein was separated by 10% SDS-PAGE and transferred onto 0.2 μm PVDF membrane and blocked with 5% nonfat dry milk in phosphate-buffered saline (PBS) containing 0.1% Tween-20 (PBS-T). Blocked membranes were exposed to primary antibodies toward total Mfn2 (Abcam mouse monoclonal anti-Mfn2, ab56889, 1:2000 dilution) and GAPDH (Cell Signaling antibodies, 36,835, 1:2000 dilution). Sheep anti-mouse immunoglobulin G (IgG) antibodies (GE Healthcare NA9310V) in 1:4000 dilution were used as secondary antibodies. Protein bands were visualized by enhanced chemiluminescence (GE Healthcare Life Sciences). Mfn2 bands were normalized to GAPDH bands using the software ScanPack 3.0.

### Real-Time PCR

After extraction of total RNA using a RNA fibrous tissue mini kit (Qiagen, Venlo, Netherlands), cDNA was synthesized from 1 μg of RNA using the iScript™ cDNA synthesis kit (Bio-Rad, Hercules, CA). Quantitative reverse transcription-polymerase chain reaction (qRT-PCR) was performed with commercial and customized TaqMan probes (Life Technologies™, Waltham, Massachusetts, USA). Target gene mRNA levels were normalized to glyceraldehyde 3-phosphate dehydrogenase (GAPDH), which served as housekeeping gene for comparison.

### Confocal Microscopy

Laser scanning confocal microscopy (Leica SP5 and a Zeiss LSM 780) was used to follow the changes in cytosolic ([Ca^2+^]_i_) and mitochondrial ([Ca^2+^]_m_) Ca^2+^ concentration, mitochondrial membrane potential (ΔΨ_m_), reactive oxygen species (ROS) generation, and mitochondrial ATP generation using specific fluorescent indicators. All fluorescent signals were recorded in electrically stimulated (0.5 Hz) single cardiac myocytes. For measurements, the myocytes were plated on laminin-covered coverslips and incubated in Tyrode solution containing 1 mM Ca^2+^ at room temperature. The fluorescence image was recorded every 10 s. Fluorescence levels were corrected for background fluorescence and normalized to untreated control (*F*_0_) (*F*/*F*_0_).

### [Ca^2+^]_m_ Measurements

Changes in mitochondrial Ca^2+^ concentration ([Ca^2+^]_m_) were measured in intact cells using the Ca^2+^-sensitive fluorescent probe X-Rhod-1/AM (molecular probes, *λ*_ex_ = 543 nm and *λ*_em_ = 552–617 nm). Myocytes were loaded with the dye for 30 min at 37°C. This loading procedure favors mitochondrial localization of X-Rhod-1. To quench the remaining cytosolic fluorescence, the myocytes were incubated in 1 mM CoCl_2_-containing Tyrode during washout period as published before (Seidlmayer et al., 2015, 2016).

### Analysis of Mitochondrial Subfractions

For the analysis of [Ca^2+^]_m_ levels in SSM, a longitudinal region of interest (ROI) was placed directly underneath the sarcolemma and for IFM in the center of the cell excluding the nuclei. Changes in [Ca^2+^]_m_ levels in perinuclear regions (PNM) were detected in the ROIs located from each side of the nucleus. Fluorescence changes over time were analyzed.

### ATP Measurement

ATP concentration was measured indirectly *via* the free magnesium (Mg^2+^) concentration using the fluorescent probe mag-fluo-4 AM (Invitrogen) and directly using a commercially available ATP luciferase assay (Invitrogen, Waltham, Massachusetts, USA, #A22066) (Seidlmayer et al., 2016). This indirect method is based on the fact that ATP forms complexes with Mg^2+^. Since free [Mg^2+^]_i_ is kept constant within a rather narrow range, ATP hydrolysis leads to concomitant increase in free [Mg^2+^]_i_ as measured with fluorescent Mg^2+^ indicators such as mag-fluo-4. Therefore, an increase in mag-fluo-4 fluorescence indicates a decrease in ATP concentration. For indirect ATP measurements, myocytes were loaded with 10 μM mag-fluo-4 (*λ*_ex_ = 488 nm and *λ*_em_ = 565–605 nm) for 30 min at 37°C. All data are expressed as an inverted ratio *R* = 1 − F/F_0_.

### Mitochondrial Membrane Potential Measurements

Mitochondrial membrane potential (Δ*Ψ_m_*) was measured in intact myocytes using the potentiometric probe tetramethylrhodamine methyl ester (TMRM) (*λ*_ex_ = 543 nm and *λ*_em_ = 565–605 nm) (Dedkova and Blatter, 2012; Seidlmayer et al., 2015, 2016). The myocytes were loaded with 10 nM TMRM for 15 min at 37°C. 10 nM TMRM was present in all solutions during the experiments. In the end of each experiment, 5 μM carbonyl cyanide p-(trifluoromethoxy)phenylhydrazone (FCCP) and 1 μM oligomycin were added to calibrate the signal. All data were background corrected.

### Redox Measurements

Isolated adult ventricular myocytes from WT mice were field-stimulated at 37°C with a stimulation rate of 0.5 Hz and superfused with normal Tyrode’s (NT) solution containing (in mM) NaCl 130, KCl 5, MgCl_2_ 1, CaCl_2_ 1, Na-HEPES 10, glucose 10, sodium pyruvate 2, and ascorbic acid 0.3, pH 7.4. After 120 s, cardiac myocytes were superfused with NT solution containing either 10 nM endothelin or 500 nM isoproterenol. We monitored changes in redox status of cardiomyocytes by measuring autofluorescence of two endogenous tissue fluorophores: reduced form of nicotinamide adenine dinucleotide (NADH, transfers electrons to molecular oxygen) and flavin adenine dinucleotide (FAD^+^, which is an electron acceptor) (Dedkova and Blatter, 2012). NADH has fluorescence excitation and emission maxima at 350 and 460 nm, respectively. FAD^+^ has fluorescence excitation and emission maxima at 450 and 535 nm, respectively. During this protocol, NADH (*λ*_ex_ = 340 nm; *λ*_em_ = 450 nm) and FAD^+^ (*λ*_ex_ = 480 nm; *λ*_em_ = 520 nm) autofluorescence were recorded using an IonOptix set-up as described previously in (Kohlhaas et al., 2017). Calibration was performed with FCCP (5 μmol/L) and cyanide (4 mmol/L). “Redox ratio,” which represents approximation of the reduction-to-oxidation status of the mitochondrial matrix space, was calculated by dividing the fluorescence intensity of NADH to the fluorescence intensity of FAD^+^.

### Statistics

**Figure 1:**
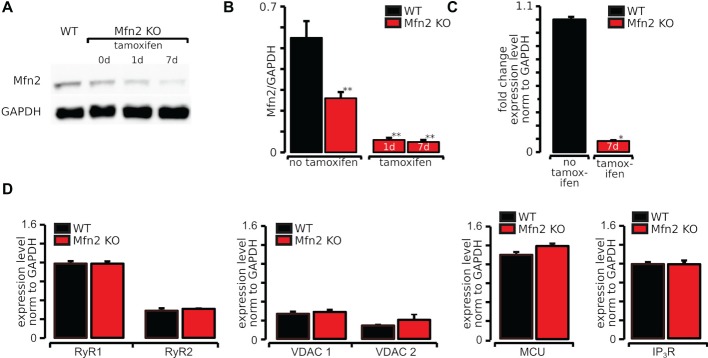
Expression of Mfn2 was effectively abolished by 7 days of tamoxifen treatment in Mfn2 KO mice. **(A)** Original Western blots of Mfn2 and GAPDH in WT and Mfn2 KO mice exposed to tamoxifen for 0, 1, and 7 days. **(B)** Mfn2 normalized to GAPDH showing a significant decrease of Mfn2 in tamoxifen-treated mice. **(C)** Fold change expression normalized to GAPDH of Mfn2 after 7 days of tamoxifen treatment compared to untreated WT measured by real-time PCR. **(D)** Expression level of RyR1, RyR2, MCU, and VDAC1 and VDAC 2 in WT and Mfn2 KO mice measured by real-time PCR. *p ≤ 0.05 compared to WT, **p ≤ 0.01 compared to WT.

The data are normalized to untreated control and background corrected. All data are presented as mean ± standard error of the mean for the indicated number (*n*) of experiments. For comparisons of multiple groups in Figure 1, ANOVA was used. A significant overall *F*-test multi-group comparison was done *via* Tukey’s test (including correction for multiple testing) for treatment vs. control. For comparisons of only two groups, Student’s *t*-test was used. Data were considered significant at *p* < 0.05.

## Results

### Expression of Mfn2 was Effectively Abolished by Tamoxifen Treatment in Mfn2 KO Mice

To test our hypothesis that disturbed SR/mitochondrial contacts due to Mfn2 knockout affect the SR/mitochondrial metabolic feedback mechanism, experiments were performed in tamoxifen-inducible cardiac-specific Mfn2 knockout (Mfn2 KO) mice (Chen et al., 2011). To verify that Mfn2 protein was effectively reduced by tamoxifen, Western blot analysis was performed in heart tissue lysates collected 1 and 7 days after beginning of tamoxifen treatment. As shown in representative Western blot images in Figure 1A, Mfn2 expression was significantly reduced after 1 day of tamoxifen treatment and almost completely eliminated (90% decrease compared to WT myocytes) after 7 days of tamoxifen (Figure 1B). These results were confirmed by quantifying RNA levels of Mfn2 using real-time PCR. As shown in Figure 1C, the relative RNA in Mfn2 KO was 0.09 ± 0.07 (*n* = 3) compared to 1.0 ± 0.37 (*n* = 4), in WT mice. To verify that knockout of Mfn2 has no effect on the proteins involved in cardiac ECC, we tested the expression levels of RyR types 1 and 2, MCU, and voltage-dependent anion channel 1 (VDAC1) and 2 (VDAC2) by real-time PCR. As demonstrated in Figure 1D, expression levels of both RyR types 1 and 2, MCU, VDAC1, and VDAC2 were not altered by Mfn2 knockout.

### Ca^2+^ Released *via* the IP_3_R Cannot Be Taken Up Into Mitochondria in Mfn2 KO mice

**Figure 2:**
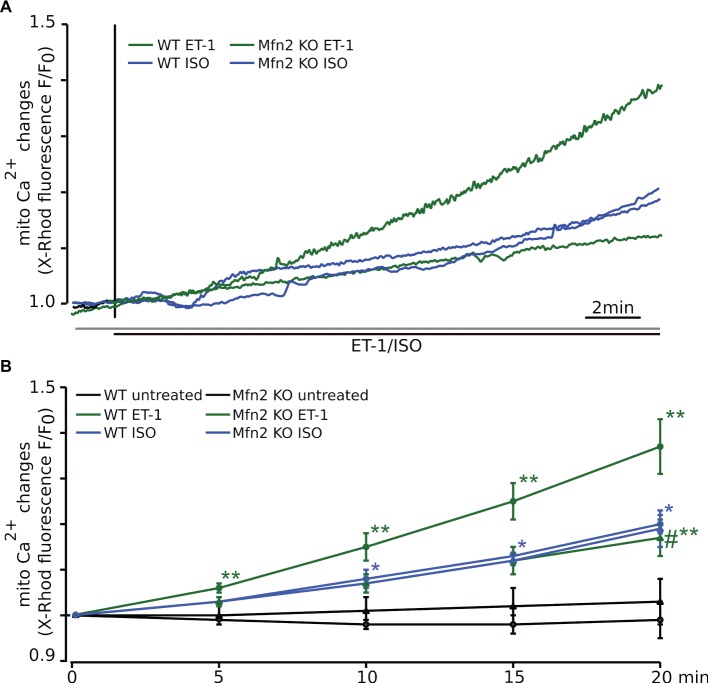
In Mfn2 KO mice Ca^2+^ released *via* the IP_3_R cannot be taken up into mitochondria. **(A)** The representative confocal traces recorded from adult ventricular myocytes loaded with the Ca^2+^-sensitive fluorescent dye X-Rhod-1 upon electrical field stimulation (FS, 0.5 Hz) and subsequent exposure to 10 nM ET-1 (WT: solid green, Mfn2 KO: dashed green) or 500 nM ISO (WT: solid blue, Mfn2 KO: dashed blue), respectively. Changes in fluorescence reflect changes in mitochondrial Ca^2+^. **(B)** Mean values of the mitochondrial Ca^2+^ levels change during 20 min of observation. ET-1 increased mitochondrial Ca^2+^ concentration ([Ca^2+^]_m_) by +35 ± 6% (*n* = 7) in healthy WT myocytes, whereas ISO-induced elevation of [Ca^2+^]_m_ was significantly smaller (+20 ± 2%, *n* = 7). In Mfn2 KO myocytes, the mitochondrial Ca^2+^ uptake induced by ET-1 was significantly smaller compared to WT myocytes (ET-1: +17 ± 4%, *n* = 9). However, the effect of ISO on mitochondrial Ca^2+^ uptake was not altered by Mfn2 KO (ISO: +19 ± 4%, *n* = 7). No significant changes in [Ca^2+^]_m_ were observed in untreated WT (solid black) and Mfn2 KO (dashed black) myocytes during 20 min of observation. *p < 0.05 compared to untreated WT, **p ≤ 0.05 compared to untreated WT, #*p* ≤ 0.05 compared to WT treated with ET-1.

We have recently demonstrated (Seidlmayer et al., 2016) that Ca^2+^ released from the SR upon IP_3_R activation is taken up into the mitochondrial matrix in WT cardiomyocytes. 10 nM ET-1 induced a significant increase in mitochondrial Ca^2+^, which could be abolished by cell treatment with 3 μM of the IP_3_R antagonist 2-APB (Seidlmayer et al., 2016). Since IP_3_-mediated global cytosolic Ca^2+^ changes are very small, experiments were designed to test the importance of the subcellular contacts between SR and mitochondria in Ca^2+^ tunneling directly from SR to mitochondria in Mfn2 KO mice in which the physical tethering of SR and mitochondria is disrupted. Consistent with our previous reports (Seidlmayer et al., 2016), addition of 10 nM ET-1 to electrically field-stimulated (0.5 Hz) ventricular myocytes induced a significant increase in mitochondrial Ca^2+^ uptake in WT myocytes (+35 ± 6%, *n* = 7, Figures 2A,B, solid green trace). This ET-1-induced mitochondrial Ca^2+^ uptake was significantly blunted in Mfn2 KO myocytes (+17 ± 4%, *n* = 9) (Figures 2A,B, dashed green trace) confirming the importance of Mfn2 in ET-1 mediated Ca^2+^ transfer to mitochondria. Furthermore, we confirmed that blocking mitochondrial Ca^2+^ uptake with the mRyR1 inhibitor dantrolene (1 μM), led to a corresponding increase in systolic Ca^2+^ level in the cytosol (Supplementary Figure S1). This indicates that when mitochondrial Ca^2+^ uptake *via* mRyR1 is not possible, Ca^2+^ released from SR is leaking out into cytosol. This Ca^2+^ leak from the SR in cytosol could contribute to pro-arrhythmic events described before (Proven et al., 2006).

Control time recordings performed in untreated electrically field-stimulated (0.5 Hz) myocytes from WT (solid black trace, −1 ± 4%, *n* = 6) and Mfn2 KO mice (dashed black trace, +3 ± 5%, *n* = 4) confirmed that there was no measurable increase in mitochondrial Ca^2+^ levels in the absence of ET-1 stimulation (Figure 2B), confirming that the observed increase in mitochondrial Ca^2+^ was due to ET-1 activation of IP_3_ receptors.

### Mfn2 KO Does Not Block Global ISO-Induced Mitochondrial Ca^2+^ Uptake

We have previously shown that when cytosolic Ca^2+^ was increased by the non-specific beta-adrenergic receptor agonist isoproterenol (ISO) in WT myocytes, which induces a global increase in cytosolic Ca^2+^ concentration, only a small increase in mitochondrial Ca^2+^ uptake was observed (Seidlmayer et al., 2016). In this study, we performed experiments in cardiomyocytes from Mfn2 KO mice, and their control littermates (WT) to examine whether intact Mfn-2-mediated contact sites are required for ISO-induced changes in [Ca^2+^]_m_. As shown in Figure 2A, we found that mitochondrial Ca^2+^ increase induced by ISO (solid blue line) in electrically field-stimulated (0.5 Hz) ventricular myocytes was significantly smaller (+20 ± 2%, *n* = 7) compared to the ET-1-induced response (solid green line). Furthermore, in contrast to the data obtained with ET-1, Mfn2 KO had no effect on average ISO-induced mitochondrial Ca^2+^ uptake (+19 ± 4%, *n* = 7, dashed blue line) (Figures 2A,B), indicating that intact spatial arrangement of SR and mitochondria was not critical for ISO-induced mitochondrial Ca^2+^ uptake.

### Interfibrillar and Perinuclear Mitochondria Take Up More Ca^2+^ Following IP_3_-Mediated SR Ca^2+^ Release Compared to Subsarcolemmal Mitochondria

**Figure 3:**
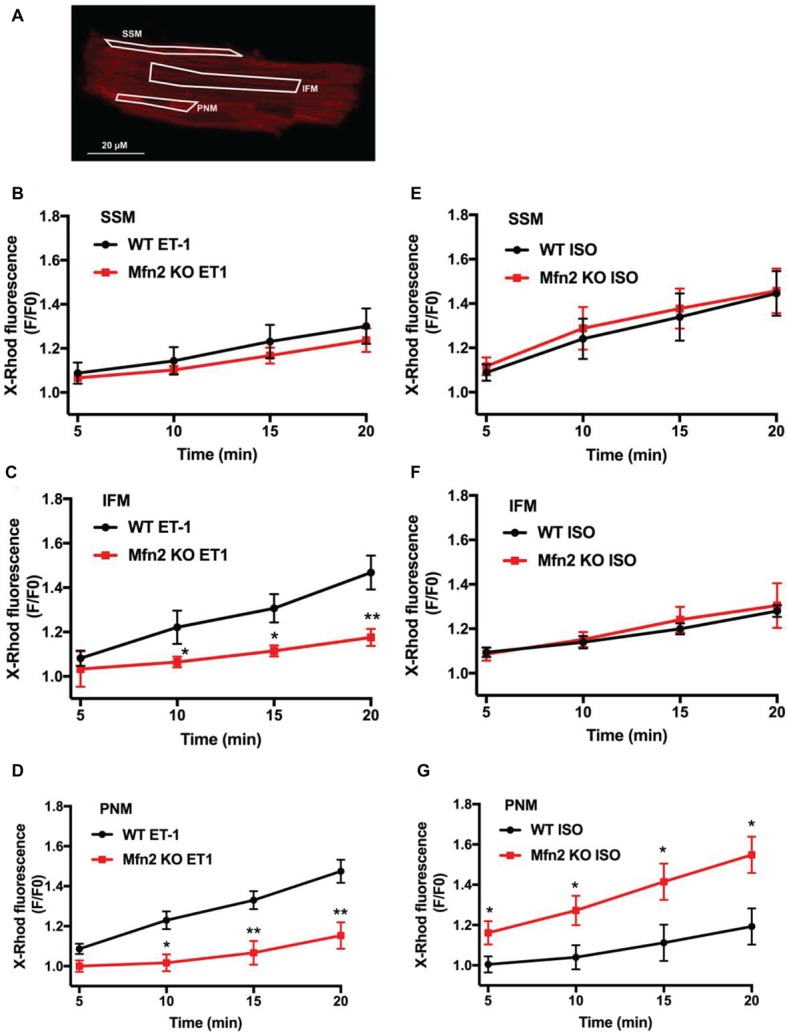
IFM and PNM take up more Ca^2+^ than SSM following IP_3_-mediated SR Ca^2+^ release. **(A)** Original confocal image of an adult murine ventricular myocyte loaded with X-Rhod-1. Shown in white are regions of interest (ROIs) placed over SSM, IFM, and PNM for the analysis of Ca^2+^ changes in different mitochondrial regions. **(B)** Mean values for mitochondrial Ca^2+^ changes measured as changes in X-Rhod-1 fluorescence in SSM normalized to untreated controls (black: WT myocytes+ET-1; red: Mfn2 KO myocytes + ET-1). **(C)** Mean values for mitochondrial Ca^2+^ changes measured as changes in X-Rhod-1 fluorescence in IFM normalized to untreated controls (black: WT myocytes+ET-1; red: Mfn2 KO myocytes + ET-1). **(D)** Mean values for mitochondrial Ca^2+^ changes measured as changes in X-Rhod-1 fluorescence in PNM normalized to untreated controls (black: WT myocytes+ET-1; red: Mfn2 KO myocytes + ET-1). **(E)** Mean values for mitochondrial Ca^2+^ changes measured as changes in X-Rhod-1 fluorescence in SSM normalized to untreated controls (black: WT myocytes+ISO; red: Mfn2 KO myocytes + ISO). **(F)** Mean values for mitochondrial Ca^2+^ changes measured as changes in X-Rhod-1 fluorescence in IFM normalized to untreated controls (black: WT myocytes+ISO; red: Mfn2 KO myocytes + ISO). **(G)** Mean values for mitochondrial Ca^2+^ changes measured as changes in X-Rhod-1 fluorescence in PNM normalized to untreated controls (black: WT myocytes+ISO; red: Mfn2 KO myocytes + ISO). *p ≤ 0.05 compared to WT ET-1 or ISO vs. Mfn2 KO, **p ≤ 0.001 compared to WT ET-1 or ISO vs. Mfn2 KO.

Since mitochondrial morphology and function vary between different mitochondrial subpopulations in the cell (Fawcett and McNutt, 1969; Palmer et al., 1977; Matlib et al., 1978; McMillin-Wood et al., 1980; Lukyanenko et al., 2009), we tested the importance of physical tethering of SR and mitochondria in different mitochondrial regions. As we mentioned before, subsarcolemmal mitochondria (SSM) exist below the cell membrane, interfibrillar mitochondria (IFM) reside in rows between the myofibrils, and perinuclear mitochondria (PNM) are located around the nuclei. Electron microscopy studies revealed that the SR/mitochondrial cleft is only 10–25 nm (Chen et al., 2012). Since, due to their intracellular localization, the interorganellar contact differs between the different mitochondrial subpopulations, we analyzed differences in IP_3_-mediated mitochondrial Ca^2+^ uptake in these mitochondrial subfractions. For the analysis of [Ca^2+^]_m_ levels in SSM, a longitudinal region of interest (ROI) was placed directly underneath the sarcolemma. For [Ca^2+^]_m_ levels in IFM, ROIs were placed in the center of the cell excluding the nuclei. Changes in [Ca^2+^]_m_ levels in perinuclear regions (PNM) were detected in the ROIs located from each side of the nucleus. As shown in Figure 3, subsarcolemmal mitochondria of WT myocytes take up significantly less Ca^2+^ than interfibrillar and perinuclear mitochondria following ET-1 stimulation (IFM: +47 ± 8% after 20 min, *n* = 6; PNM: +47 ± 6% after 20 min, *n* = 6 vs. SSM: +30 ± 8% after 20 min, *n* = 6). In Mfn2 KO mice, mitochondrial Ca^2+^ uptake was significantly inhibited in IFM (+24 ± 4%, *n* = 6, *p* < 0.05 compared to WT) and PNM (+19 ± 4%, *n* = 6, *p* < 0.05) mitochondria following ET-1 stimulation. There was no difference in [Ca^2+^]_m_ levels upon ET-1 treatment in Mfn2 KO mitochondria from SSM regions (+20 ± 2%, *n* = 8, *p* = 0.23) (Figure 3). These data indicate that disruption of the SR-mitochondrial tethering in specific regions abolishes the differences in mitochondrial Ca^2+^ uptake induced by IP_3_-mediated Ca^2+^ release from SR in ventricular myocytes.

Interestingly, the effect of ISO on [Ca^2+^]_m_ levels in the three mitochondrial subgroups was different from ET-1. The highest mitochondrial Ca^2+^ uptake was detected in subsarcolemmal mitochondria (+44 ± 12% after 20 min, *n* = 7), which gradually declined in IFM (+20 ± 9% after 20 min, *n* = 7) and was the smallest in perinuclear (+11 ± 9% after 20 min, *n* = 7) mitochondria. Furthermore, while Mfn-2 KO had no effect on ISO-induced [Ca^2+^]_m_ levels in SSM and IFM mitochondrial regions (Figures 3E,F), mitochondrial Ca^2+^ uptake was actually increased in perinuclear mitochondria (Figure 3G) in cardiomyocytes from Mfn2 KO animals.

These data suggest that tethering between SR and mitochondria is heterogeneous within ventricular myocytes to support the privileged SR-mitochondrial communication between IP_3_ receptors and mRyR1. When this communication was lost in Mfn-2 KO mice, perinuclear mitochondria were able to pick more Ca^2+^ upon ISO stimulation.

### IP_3_-Mediated Increase in Mitochondrial Membrane Potential was Dependent on Mfn2

**Figure 4:**
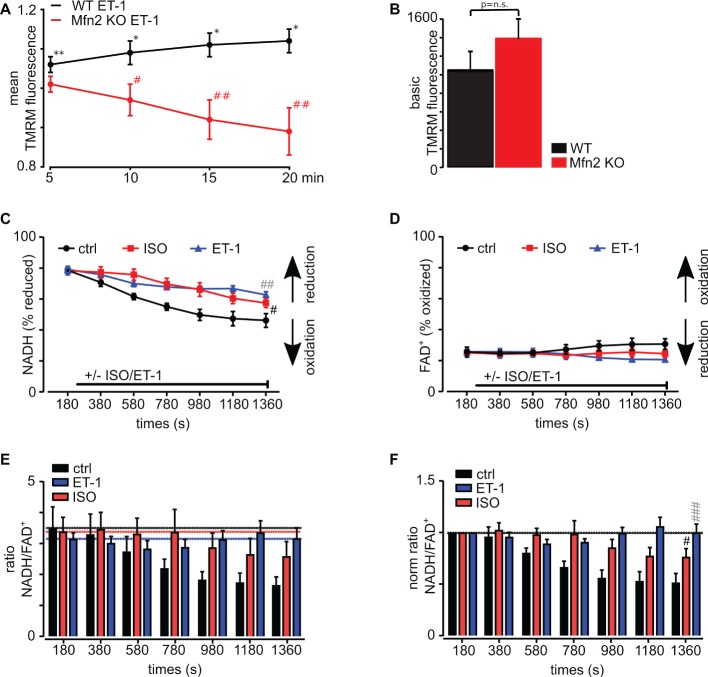
IP_3_R stimulation with ET-1 increases mitochondrial membrane potential in WT myocytes but not in Mfn2 KO myocytes. **(A)** Normalized mean values of TMRM fluorescence changes in WT (black line) and Mfn2 KO (red line) cardiac myocytes after exposure to 10 nM ET-1 for 20 min. TMRM signal was normalized to the level measured in untreated WT and Mfn2 KO myocytes. **(B)** Mean values of basal TMRM fluorescence in untreated myocytes from WT (black) and Mfn2 KO (red) mice. **(C)** Mean values of NADH in % of reduced NADH following ISO (red) and ET-1 (blue) treatment as well as untreated control (black) over 20 min. **(D)** Mean values of FAD^+^ in % of oxidized FAD^+^ following ISO (red) and ET-1 (blue) treatment as well as untreated control (black) over 20 min. **(E)** The ratio of NADH/FADH following the treatment with ET-1 (blue) and ISO (red). For comparison, untreated control is shown in black. **(F)** The normalized ratio of NADH/FADH following treatment with ET-1 (blue) and ISO (red) is shown. Black is the untreated control with electrical field stimulation at frequency 0.5 Hz. **(A)** *p ≤ 0.05 compared to untreated control, **p ≤ 0.001 compared to untreated control, #*p* ≤ 0.05 compared to WT treated with ET-1, ##*p* ≤ 0.001 compared to WT treated with ET-1. **(C–F)** #*p* ≤ 0.05, ##*p* ≤ 0.01, ###*p* ≤ 0.001; black – ISO vs. ctrl, gray – ET-1 vs. ctrl.

The Ca^2+^ taken up by mitochondria following ET-1 stimulation is activating mitochondrial oxidative phosphorylation, which results in a significant increase in ATP levels (Seidlmayer et al., 2016). Here, we measured changes in mitochondrial membrane potential (Δ*Ψ_m_*) following ET-1 stimulation using the potentiometric probe TMRM in cardiac myocytes from WT and Mfn2 KO mice (Figure 4A). In WT myocytes, ET-1 induced a significant hyperpolarization over 20 min (+37 ± 3%, *n* = 13, *p* < 0.01 compared to untreated controls), whereas in Mfn-2 KO Δ*Ψ_m_* decreased upon exposure to ET-1 (−11 ± 6%, *n* = 4, *p* < 0.01 compared to WT + ET-1) myocytes, indicating the importance of intact SR-mitochondrial contacts for mitochondrial Δ*Ψ_m_* maintenance.

To test if basal Δ*Ψ_m_* was different in WT and Mfn2 KO myocytes, we measured TMRM fluorescence at the beginning of the experiment before cell treatment with ET-1. No significant difference in basal Δ*Ψ_m_* was detected (Figure 4B), suggesting that there is no difference in basal respiratory activity in WT and Mfn2 KO mice.

In support of these data, we also found that while both ET-1 and ISO induced a decrease in reduced NADH (Figure 4C), changes in both reduced NADH and oxidized FAD^+^ levels (Figure 4D) were less pronounced upon ET-1 stimulation resulting in minimal net redox ratio NADH/FAD^+^ changes (Figures 4E,F) normalized to untreated conditions whereas, in cells treated with ISO, the ratio declined significantly. Altogether, these data indicate that ET-1 maintains mitochondrial membrane potential and respiratory chain activity.

### IP_3_-Mediated ATP Production Following ET-1 Stimulation is Impaired in Mfn2 KO Mice

**Figure 5:**
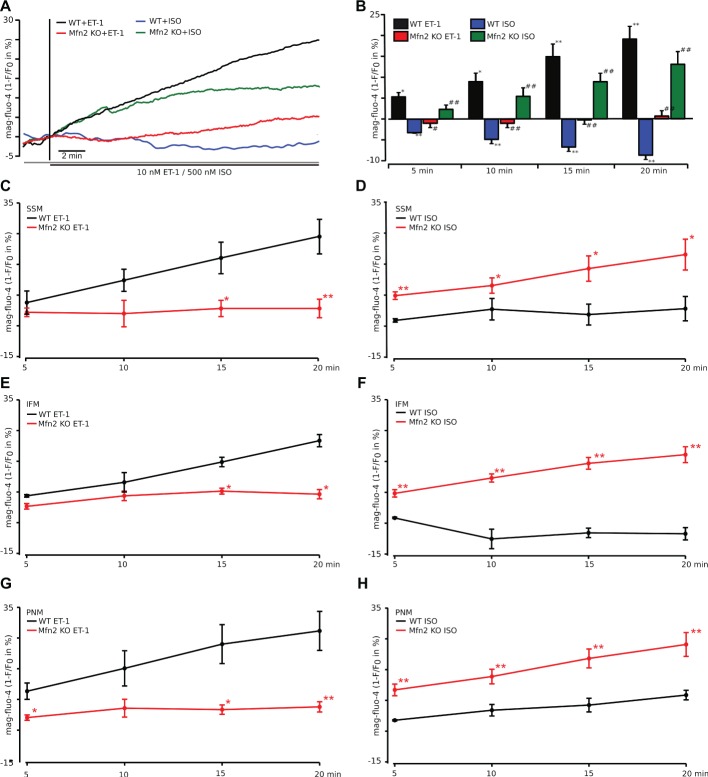
ATP production following IP_3_-mediated SR Ca^2+^ release is impaired in Mfn2 KO mice. **(A)** Original confocal traces of ATP levels recorded in WT myocytes stimulated with 10 nM ET-1 (black) or 500 nM ISO (blue) and Mfn2 KO cardiac myocytes stimulated with ET-1 (red) or ISO (green), respectively. Mg^2+^-sensitive fluorescent dye mag-fluo-4 was used to monitor ATP levels inside cardiac myocytes. As free Mg^2+^ and ATP form complexes, increase in ATP concentration reduces free Mg^2+^ and thus mag-fluo-4 fluorescence. Traces are inverted for better understanding and presented as 1-F/F_0_. **(B)** Mean values of the experiment described in **(A)** in %. *p ≤ 0.05 compared to untreated WT; **p ≤ 0.01 compared to untreated WT; #*p* ≤ 0.05 compared to WT treated with ET-1 or ISO, respectively; ##*p* ≤ 0.01 compared to WT treated with ET-1 or ISO, respectively. **(C–H)** Subgroup analysis of subsarcolemmal (SSM, **C,D**), interfibrillar (IFM, **E,F**), and perinuclear (PNM, **G,H**) mitochondria in myocytes from WT (black) and Mfn2 KO (red) mice loaded with mag-fluo-4 as described in **(A)**. The myocytes were treated with either ET-1 (left) or ISO (right). *p ≤ 0.05 compared to WT, **p ≤ 0.01 compared to WT.

Next, we monitored changes in [ATP]_i_ indirectly using the [Mg^2+^]-sensitive dye mag-fluo-4 in WT and Mfn-2 KO cardiac myocytes treated with either 10 nM ET-1 or 100 nM ISO. This method takes advantage of the fact that ATP is a major Mg^2+^ buffer, such that an increase in [ATP]_i_ level reduces free [Mg^2+^]_i_ (Dedkova and Blatter, 2012). Therefore, increased ATP production causes mag-fluo-4 fluorescence to decline. In Figure 5, the mag-fluo-4 signal was inverted so that an increase in the signal reflects an increase in [ATP]_i_. Similar to previous experiments, cardiac myocytes were electrically stimulated at the frequency of 0.5 Hz to maintain Ca^2+^ fluxes during ECC, and then ET-1 (10 nM) and ISO (500 nM) were applied to activate the correspondent receptors. As shown in Figure 5A, ET-1 but not ISO induced an increase in ATP generation in WT ventricular myocytes, which was abolished in Mfn2 KO myocytes emphasizing the importance of Mfn2 in Ca^2+^ transfer to mitochondria to mediate ATP production. As summarized in Figure 5B, ET-1 induced a significant increase in ATP levels over 20 min (+19.18 ± 3.7%, *n* = 6), whereas in Mfn2 KO myocytes the effect of ET-1 on ATP generation was completely abolished (+0.74 ± 1.5%, *n* = 5). To the contrary, ISO-treatment caused a significant decrease in ATP levels in WT (−8.7 ± 1.2%, *n* = 4, *p* < 0.01), while a significant increase in ATP (+13.1 ± 3%, *n* = 7, *p* < 0.01) was observed in Mfn-2 KO myocytes upon ISO stimulation.

We further analyzed the effect of ET-1 and ISO on ATP content in mitochondrial subgroups as we did for mitochondrial Ca^2+^ uptake. The results are summarized in Figure 5C. As expected, in Mfn2 KO myocytes, ET-1 was not able to induce a significant increase in ATP levels in all subgroups. However, there was no significant difference in ET-1-induced ATP increase between IFM, SSM, and PNM in myocytes from WT or Mfn2 KO mice.

A decrease in ATP levels upon ISO exposure was more pronounced in IFM and SSM mitochondrial subgroups but remained unchanged in PNM (Figure 5C). In agreement, with the data presented in Figure 5B, ISO addition to Mfn2 KO myocytes increased ATP levels in all groups significantly compared to the corresponding groups in WT myocytes. Furthermore, PNM in Mfn 2 KO myocytes, which took more Ca^2+^ into the mitochondria, also increased ATP levels significantly higher than IFM and SSM from Mfn2 KO myocytes.

## Discussion

In this study, we examined the role of SR-mitochondrial tethering by Mfn2 in activation of mitochondrial Ca^2+^ uptake and metabolism using mice with a cardiac-specific deletion of Mfn2 (Figure 1). We tested the hypothesis that physical linkage of SR and mitochondria *via* Mfn2 is essential for IP_3_R-mediated signal transduction to mitochondria to maintain ATP levels in cardiac myocytes under physiological conditions (i.e., normal levels of IP_3_R expression and circulating ET-1). In our previous study (Seidlmayer et al., 2016), we found that the stimulation of cardiac myocytes with IP_3_R agonists induced mitochondrial Ca^2+^ uptake *via* mRyR1 and increased ATP content. In contrast, under basal conditions, beta-adrenergic stimulation with ISO was not able to induce measurable ATP production. Since the global changes in cytosolic Ca^2+^ concentration produced by IP_3_-mediated SR Ca^2+^ release are very small compared to RyR-mediated Ca^2+^ changes, here, we examined the significance of intact SR-mitochondrial tethering by Mfn2 for IP_3_-mediated stimulation of mitochondrial Ca^2+^ uptake and ATP generation.

**Figure 6:**
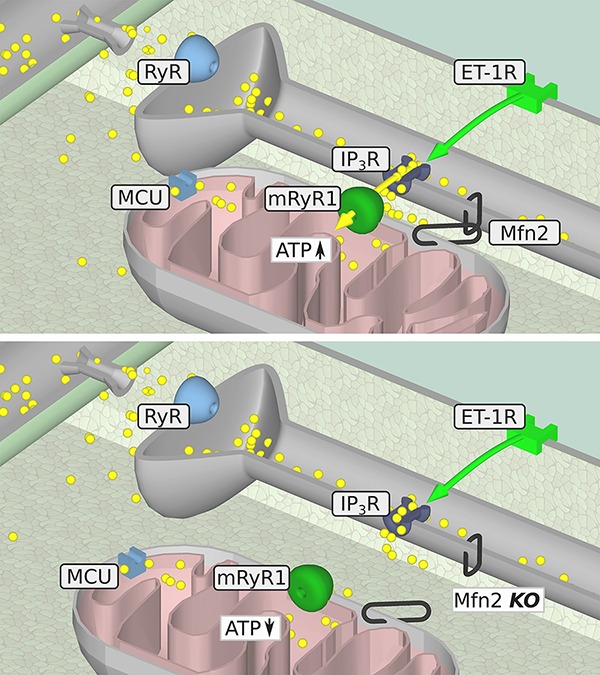
Summary of the proposed metabolic IP_3_-mediated SR/mitochondrial signaling pathway in the presence (upper panel) or absence (lower panel) of intact Mfn2-tethers. IP_3_-mediated mitochondrial Ca^2+^ uptake in WT myocytes (upper panel), which is followed by an increase in ATP generation. When the physical contact of mitochondria and the SR is disturbed by knockout of Mfn2 (lower panel), Ca^2+^ released from the SR by IP_3_ cannot be taken up into mitochondria anymore leading to the decrease in ATP level. Yellow circles: Ca^2+^, yellow arrow: Ca^2+^ movement, green arrow: IP_3_ movement.

We found that the ability of ET-1 to induce a significant IP_3_-dependent mitochondrial Ca^2+^ uptake was lost in myocytes from Mfn2 KO mice (Figure 2). Interestingly, when myocytes from Mfn2 KO mice were stimulated with the beta-adrenergic receptor agonist ISO, mitochondrial Ca^2+^ uptake was not affected in SSM and IFM but significantly increased in PNM regions. When the influence of beta-adrenergic and IP_3_R stimulation on ATP content was examined (Figure 5), ET-1 did not induce an increase in ATP in myocytes from Mfn2 KO mice, indicating the importance of physical tethering of mitochondria and the SR for metabolic control by IP_3_ under physiological conditions. To the contrary, in Mfn2 KO myocytes, ISO significantly increased ATP content under conditions of electrical field stimulation, whereas in WT myocytes ISO led to a decrease in mitochondrial ATP content. The latter finding agrees with previous studies showing that sudden increase in work load causes a decrease in mitochondrial NADH (and presumably ATP levels), which recovered slowly over the time in a Ca^2+^-dependent manner (Brandes and Bers, 1997). Furthermore, another study demonstrated that beta-adrenergic stimulation with ISO induced only a transient MCU-dependent increase in oxygen consumption, suggesting that MCU only plays a role in acute matching of the mitochondrial energy output with increase in cardiac metabolic demand (Kwong et al., 2015). However, over the time, oxygen consumption was not different between WT and MCU KO cardiac myocytes, indicating that another mitochondrial Ca^2+^ influx pathway contributes to the sustained energy generation in cardiac myocytes. Our data demonstrate that IP_3_R-mRyR1-mediated mitochondrial Ca^2+^ uptake contributes to the sustained ATP generation (Figure 6) and that this Ca^2+^ transfer from SR is regulated by Mfn2 under physiological conditions. When this regulation is lost in Mfn2 KO mice, IP_3_-mediated Ca^2+^ release is not capable to stimulate ATP generation anymore, as observed in pathological conditions. Altogether, our data suggest that Mfn2 can provide a fine tuning to regulate Ca^2+^-dependent energy production under physiological conditions upon ET-1 stimulation, but when Mfn2-mediated tethering is disrupted, mitochondria can adapt to respond to global Ca^2+^ elevation induced by ISO with increase in ATP generation.

The disruption of Mfn2-mediated intact ER-mitochondrial contacts exists in conditions of heart failure induced by transverse aortic constriction (TAC), which is associated with a decreased excitability and force generation in cardiac myocytes (Goh et al., 2016). ET-1 levels are typically increased in chronic stress as in the setting of congestive heart failure (CHF) (Wei et al., 1994) along with increased myocardial expression of ET_A_ receptors (Picard et al., 1998; Pieske et al., 1999). To determine if this increase in the ET-1 axis is pathologic, a mouse model was developed to conditionally overexpress ET-1 in the myocardium that led to nearly 10-fold increase in ET-1 concentration in the heart, without any significant increase in circulating ET-1 levels (Yang et al., 2004). Mice with elevated cardiac ET-1 expression presented with severe heart inflammation, hypertrophy leading to dilated cardiomyopathy, CHF, and death within 5 weeks of induction. However, a whole body ET-1 KO (ET-1^−/−^) homozygous mice developed by disrupting exon 2 of the ET-1 gene were neonatally lethal (Kurihara et al., 1994). ET-1^−/−^ mice delivered by caesarean section at day 18.5 postcoitum, all displayed significant craniofacial and cardiac abnormalities, highlighting the critical role of ET-1 in the development (Kurihara et al., 1994, 1995b). Moreover, ET-1^−/−^ mice had reduced neonatal weight, impaired thyroid and thymus development (Kurihara et al., 1995a), and reduced cardiac sympathetic innervation (Ieda et al., 2004). Mice heterozygous for ET-1 deletion, ET-1^+/−^, appeared normal, were fertile, and had reduced lung and plasma ET-1 concentration; however, they had elevated systolic, diastolic, and mean arterial blood pressure (Kurihara et al., 1994). These observations suggest that while 10-fold elevation in ET-1 levels in cardiac myocytes is clearly pathological, basal levels of ET-1 are required for maintaining normal physiological function in cardiac myocytes.

Indeed, myocardial ET-1 depicts beneficial effects depending on the physiological situation. In the myocardium, ET-1 prevents excessive apoptosis after cardiac overload induced by aortic banding (Zhao et al., 2006). Signaling of the anti-apoptotic effects of ET-1 involves calcineurin, the mitochondrial function, and the classical MEK1/2-ERK1/2 and PI3 kinase pathways (Iwai-Kanai and Hasegawa, 2004). It has been reported that ET-1 prevents the early phase of doxorubicin-induced cytotoxicity *via* the upregulation of the antioxidant manganese superoxide dismutase through the ET_A_ receptor in cultured cardiomyocytes (Suzuki and Miyauchi, 2001). Preconditioning infusion of ET-1 can reduce infarct size in rats subjected to ischemia-reperfusion (Gourine et al., 2005). The transgenic expression of ET-1 in mice lacking a functional gene for eNOS restores diastolic function presumably through modulation of oxidative stress and a change of metabolic substrate from fatty acid oxidation toward enhanced glycolysis (Vignon-Zellweger et al., 2011). In line with this, the enhanced glycolysis in the failing heart, which is a beneficial compensatory mechanism, is accompanied by a hypoxia-inducible factor-1α-dependent elevation of ET-1 expression (Kakinuma et al., 2001).

Our study indicates that stimulation of ET-1 receptors in healthy control myocytes leads to mitochondrial Ca^2+^ uptake, maintains mitochondrial membrane potential, and activates ATP generation. Furthermore, we determined that intact Mfn2 tethers were required to mediate Ca^2+^ uptake into mitochondria and stimulate ATP production. The significance of a physical linkage of SR and mitochondria in cardiac myocytes is still debated. Our data add new aspects to this discussion revealing the importance of Mfn2 tethers in IP_3_-mediated SR/mitochondrial metabolic feedback in ventricular myocytes. As shown by our group before (Seidlmayer et al., 2016), Ca^2+^ released from the SR *via* the IP_3_R is taken up into mitochondria where it increases ATP content. Here, we were able to demonstrate that this metabolic feedback is severely disturbed in Mfn2-deficient mice. When cardiac myocytes from these mice were stimulated with the IP_3_R agonist ET-1, no mitochondrial Ca^2+^ uptake could be detected. In agreement with this finding, IP_3_-mediated SR Ca^2+^ release in Mfn2-deficient myocytes was not able to increase cellular ATP content. These results clearly show that intact tethering of SR and mitochondria *via* Mfn2 is crucial for the channeling of intracellular Ca^2+^ signals from IP_3_R to mRyR1 located in mitochondria. In support of this, data presented in Supplementary Figure S1 demonstrate that when cells were treated with the mRyR1 inhibitor, 1 μM Dantrolene, the systolic Ca^2+^ level in cytosol was significantly elevated.

It is generally accepted that Ca^2+^ crosses the outer mitochondrial membrane *via* non-specific high-conductance voltage-dependent anion channels (VDAC) (see Shoshan-Barmatz et al., 2008, for review). Specifically, it has been demonstrated that VDAC1 plays a major role in ER/mitochondria-Ca^2+^ cross-talk (Shoshan-Barmatz et al., 2017) by forming a supra-molecular complex with the IP_3_ receptor in the SR and VDAC1 in the OMM, linked by a chaperone called GRP75 (Csordás et al., 2006), together with Mfn2 (de Brito and Scorrano, 2008). It is plausible that Mfn2-mediated tethering creates a microenvironment with highly elevated Ca^2+^ levels to enhance Ca^2+^ transfer *via* VDAC1; however, the direct experimental evidence of the enhanced Ca^2+^ channeling *via* VDAC1 by Mfn2 is lacking. Several studies, however, demonstrated that VDAC1 is highly Ca^2+^-permeable and modulates the accessibility of Ca^2+^ to the intermembrane space (IMS) (Gincel et al., 2001; Rapizzi et al., 2002; De Stefani et al., 2012). The importance of the 18-kDa outer mitochondrial membrane transporter protein (TSPO) in mitochondrial Ca^2+^ transport and its role in heart failure have been recently revealed by Thai et al. (2018) ringing attention to the outer mitochondrial membrane proteins in regulation of the mitochondrial Ca^2+^ uptake and bioenergetics.

Interestingly, not all mitochondria within the myocyte take up Ca^2+^ into their matrix following IP_3_R activation to the same extent. Detailed analysis of mitochondrial subpopulations revealed that in WT cardiac myocytes IFM and PNM take up almost two times more Ca^2+^ following IP_3_-mediated SR Ca^2+^ release compared to the SSM regions (Figure 3). In Mfn2-deficient myocytes, mitochondrial Ca^2+^ uptake following IP_3_R activation was not different between all three mitochondrial subpopulations. This finding is supported by several studies providing evidence for not only structural differences of IFM (Fawcett and McNutt, 1969; Shimada et al., 1984; Lukyanenko et al., 2009), PNM, and SSM but also functional differences between these mitochondrial populations. It has been suggested that IFM produce ATP primarily for contraction, PNM for nuclear processes, and SSM provide ATP for active transport processes across the sarcolemma (Müller, 1976; Palmer et al., 1977; Shimada et al., 1984; Rosca and Hoppel, 2013). Since ET-1 has a positive inotropic effect and activates pathological gene transcription in the nucleus, a preferential effect of ET-1 on IFM and PNM is in agreement with these findings.

As expected, since mitochondrial Ca^2+^ uptake following ET-1 stimulation was abolished in Mfn-2 KO mice, ATP production was not detectable in Mfn2-deficient cardiomyocytes. Interestingly, the basal mitochondrial membrane potential measured with TMRM was not different between the two genotypes, which is in accordance with the results of Chen et al. (2012); however, ET-1 stimulation led to the increase in mitochondrial membrane potential (Figure 4), which contributes to the enhanced electron transfer chain coupling and ATP generation (Figure 5). Taken together, these results indicate a crucial role of physical tethering of SR and mitochondria by Mfn2 for adaptation of energy production to chronic stress. Interestingly, the effect of ISO on [Ca^2+^]_m_ levels in the three mitochondrial subgroups was different from ET-1. The highest mitochondrial Ca^2+^ uptake was detected in subsarcolemmal mitochondria, which gradually declined in IFM and was the smallest in perinuclear mitochondria. Furthermore, while Mfn-2 KO had no effect on ISO-induced [Ca^2+^]_m_ levels in SSM and IFM mitochondrial regions (Figures 3E,F), mitochondrial Ca^2+^ uptake actually increased in perinuclear mitochondria (Figure 3G) in cardiomyocytes from Mfn2 KO animals.

These data suggest that tethering between SR and mitochondria is heterogeneous within ventricular myocytes to support the privileged SR-mitochondrial communication between IR3 receptors and mRyR1. When this communication was lost in Mfn-2 KO mice, perinuclear mitochondria picked up significantly more Ca^2+^ upon ISO stimulation. This increase in mitochondrial Ca^2+^ during ISO stimulation in PNM regions in Mfn-2 KO mice was also associated with the highest ATP levels in Mfn-2 deficient myocytes (see Figure 5). This difference between ET-1 and ISO responses in WT and Mfn-2 KO mice could explain the existent controversy in the field [(Filadi et al., 2015) vs. (de Brito and Scorrano, 2008) and (Naon et al., 2016)] on the role of Mfn2-mediated tethers in SR-mitochondrial Ca^2+^ communication.

## Conclusions

Our study demonstrates that in ventricular myocytes, basal ET-1 signaling is required for maintaining normal cardiac function and bioenergetics (Figure 6). We have shown that when the physical linkage between SR and mitochondria Mfn-2 is disrupted, the SR/mitochondrial metabolic feedback mechanism is severely impaired resulting in the inability of the IP_3_-mediated SR Ca^2+^ release to induce ATP production. These findings could explain the impaired cardiac function observed in aged mice and young mice following TAC (Goh et al., 2016) where Mfn2 levels were decreased leading to an impaired metabolic IP_3_-mediated SR/mitochondrial feedback.

## Ethics Statement

This study was carried out in accordance with the recommendations of the National Institutes of Health (NIH Publication NO. 85-23, revised 1996). The protocol was approved by the University of Würzburg Institutional Animal Care and Use Committee.

## Author Contributions

LS and ED designed the study and wrote the manuscript. LS, CMag, AB, and MKa performed the experiments. LS, CMag, MKa, and ED analyzed the data. MS and GD provided Mfn 2 KO mice. PE-N and PA-L supported equipment. ED, LS, BG, CMaa, and MKo edited the manuscript. BG and SF provided financial support.

### Conflict of Interest Statement

The authors declare that the research was conducted in the absence of any commercial or financial relationships that could be construed as a potential conflict of interest.

## Supplementary Material

The Supplementary Material for this article can be found online at: https://www.frontiersin.org/articles/10.3389/fphys.2019.00733/full#supplementary-material

## Abbreviations

Δ*Ψ_m_*: Mitochondrial membrane potential
ACE: Angiotensin converting enzyme
Ang II: Angiotensin II
Ca^2+^: Calcium
[Ca^2+^]_i_: Cytosolic calcium concentration
[Ca^2+^]_m_: Mitochondrial calcium concentration
CHF: Congestive heart failure
ECC: Excitation-contraction coupling
ET-1: Endothelin-1
FAD: Flavin adenine dinucleotide
IFM: Interfibrillar mitochondria
IMM: Inner mitochondrial membrane
IP_3_: Inositol 1,4,5-trisphosphate
IP_3_R: Inositol 1,4,5-trisphosphate receptor
ISO: Isoproterenol
KO: Knockout
MCU: Mitochondrial Ca^2+^ uniporter
Mfn2: Mitofusin 2
mRyR1: Mitochondrial ryanodine receptor type 1
NADH: Reduced nicotinamide adenine dinucleotide
PNM: Perinuclear mitochondria
RyR1: Ryanodine receptor type 1
SR: Sarcoplasmic reticulum
SSM: Subsarcolemmal mitochondria
VDAC1: Voltage-dependent anion channel 1
VDAC2: Voltage-dependent anion channel 2
WT: Wild-type.
