# Supplementary material for: Mitofusin 2 Is Essential for IP3-Mediated SR/Mitochondria Metabolic Feedback in Ventricular Myocytes
Source: Front Physiol. 2019 Jul 18;10:733. doi: 10.3389/fphys.2019.00733 (PMC6658196; doi:10.3389/fphys.2019.00733)
Supplement: Supplementary file 1 [file Data_Sheet_1.PDF]

Supplemental Materials

Methods

Cytosolic Ca^{2+} measurements

Cytosolic Ca^{2+} levels (Ca^{2+}_i) were measured during distole and systole using fluorescent dye Fluo-4. For Ca^{2+}_i measurements, cardiac myocytes were loaded with 20 µM of the Ca^{2+} sensitive dye fluo-4 (λ_{ex} = 488 nm and λ_{em} = 565–605 nm) for 20 min at room temperature. To block the mRyR1, cells were pre-incubated in 1 µM Dantrolene for 10 min, and 1 µM Dantrolene was present during the experiment. Changes in Ca^{2+} concentration are presented as relative changes in fluorescence because the calibration of non-ratiometic dyes used in our study in terms of Ca^{2+} concentration is not possible. For easier understanding of the manuscript, the relative changes in fluorescence measured by us will be termed "Ca^{2+} concentration" throughout the manuscript.

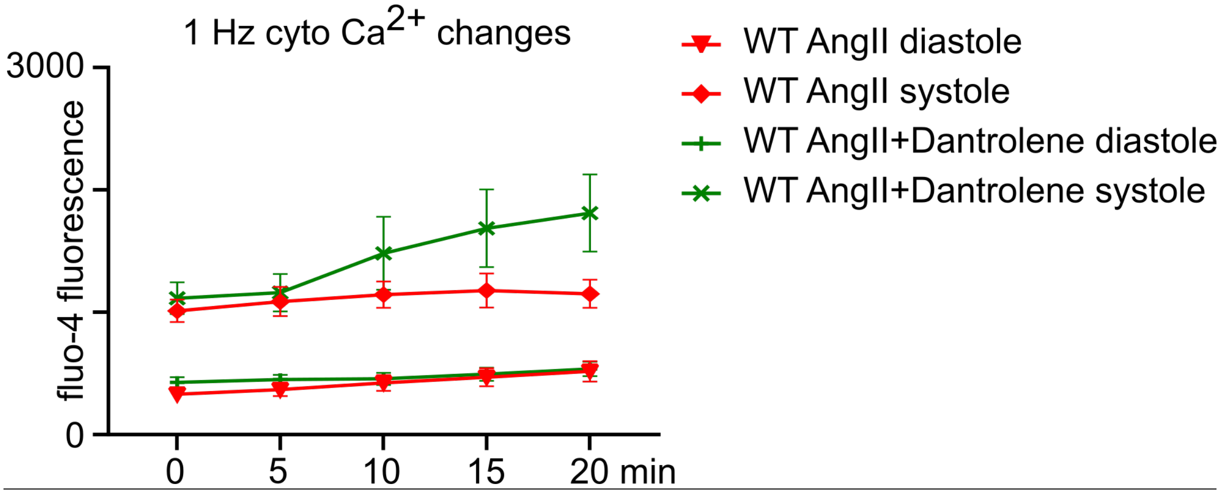

Figure S1. Mean values of cardiomyoytes treated with 2 µM AngII in the absence (red) or presence of 1 µM mRyR1 blocker Dantrolene (green). To block the mRyR1 cells were pre-incubated in 1 µM Dantrolene for 10 min, and 1 µM Dantrolene was present during the experiment. Cells were electrically-field stimulated at 1 Hz and changes in diastolic and systolic cytosolic Ca^{2+} levels were measured over 20 min.
